# *Mycobacterium tuberculosis* Load in Host Cells and the Antibacterial Activity of Alveolar Macrophages Are Linked and Differentially Regulated in Various Lung Lesions of Patients with Pulmonary Tuberculosis

**DOI:** 10.3390/ijms22073452

**Published:** 2021-03-26

**Authors:** Elena G. Ufimtseva, Natalya I. Eremeeva, Tatiana V. Umpeleva, Diana V. Vakhrusheva, Sergey N. Skornyakov

**Affiliations:** 1 Laboratory of Medical Biotechnology, Research Institute of Biochemistry, Federal Research Center of Fundamental and Translational Medicine, 2 Timakova Street, 630117 Novosibirsk, Russia; 2 Scientific Department, Ural Research Institute for Phthisiopulmonology, National Medical Research Center of Tuberculosis and Infectious Diseases of Ministry of Health of the Russian Federation, 50 XXII Partsyezda Street, 620039 Yekaterinburg, Russia; eremeevani@yandex.ru (N.I.E.); tumpeleva@ya.ru (T.V.U.); vakhrusheva@urniif.ru (D.V.V.); sns@urniif.ru (S.N.S.)

**Keywords:** *Mycobacterium tuberculosis*, patients with pulmonary tuberculosis, alveolar macrophages, lung tuberculous lesions, immune response, inflammation, fibrosis

## Abstract

Tuberculosis (TB) is a disease caused by *Mycobacterium tuberculosis* (*Mtb*) infection with the formation of a broad range of abnormal lung lesions within a single patient. Although host–pathogen interactions determine disease outcome, they are poorly understood within individual lesions at different stages of maturation. We compared *Mtb* load in a tuberculoma wall and the lung tissue distant from tuberculomas in TB patients. These data were combined with an analysis of activation and bactericidal statuses of alveolar macrophages and other cell subtypes examined both in ex vivo culture and on the histological sections obtained from the same lung lesions. The expression of pattern recognition receptors CD14, CD11b, and TLR-2, transcription factors HIF-1α, HIF-2α, and NF-*κ*B p50 and p65, enzymes iNOS and COX-2, reactive oxygen species (ROS) biosynthesis, and lipid production were detected for various lung lesions, with individual *Mtb* loads in them. The walls of tuberculomas with insufficient inflammation and excessive fibrosis were identified as being the main niche for *Mtb* survival (single or as colonies) in non-foamy alveolar macrophages among various lung lesions examined. The identification of factors engaged in the control of *Mtb* infection and tissue pathology in local lung microenvironments, where host–pathogen relationships take place, is critical for the development of new therapeutic strategies.

## 1. Introduction

Tuberculosis (TB), which is caused by the pathogen *Mycobacterium tuberculosis* (*Mtb*), remains the leading bacterial infectious cause of morbidity and mortality worldwide [1]. Today, an increasingly rapid spread of multidrug-resistant (MDR) TB is one of the main barriers obstructing the treatment of TB [2,3]. *M. tuberculosis* is usually transmitted via aerosols produced by a sputum-positive person with active TB and inhaled into the alveolar space of a new host, where these bacteria are engulfed by alveolar macrophages [4,5]. In human lungs, the *Mtb*-associated molecules such as the 38-kDa phosphate binding glycoprotein PstS-1, known as the 38-kDa antigen (Ag38), and the lipoarabinomannan (LAM) interact with the pattern recognition receptors (PRRs) on the immune cells and initiate intracellular signaling cascades, leading to the generation of reactive oxygen species (ROS), and activation of the nuclear factor-kappa B (NF-*κ*B) and hypoxia-inducible factor (HIF) signaling pathways, and ultimately to the inflammatory and bactericidal responses against invading pathogens [6,7,8,9,10,11,12].

An excessive and prolonged inflammatory response results in the recruitment and accumulation of immune cells at the site of the *Mtb* infection in the alveoli and triggers the formation of multiple injuries in human lungs [4,5]. The TB lung lesions include complex organized structures known as granulomas [4,5,8,13] and local or extensive tissue remodeling known as fibrosis [13,14,15]. Within the same lungs of patients with pulmonary TB, there are multiple and structurally (morphologically and phenotypically) diverse types of granulomatous lesions. They range from small immune cell aggregations without proper necrotic formation and fibrotic encapsulated nodules (about 1–5 mm in diameter on average) with a caseous center and a dense peripheral leukocyte rim to larger and more differentiated tuberculomas (more than 12 mm in diameter) with a thicker dense wall and a big mass of central necrotic debris that may undergo liquefaction resulting in the formation of large open cavities connected with an airway [13,16,17]. Acid-fast *Mtb*-positive sputum samples are mainly detected for patients with open cavitary TB [16,17,18,19], which facilitates *Mtb* dissemination via coughing.

Histological examination of the TB patients’ lung lesions revealed a heterogeneous distribution of acid-fast *Mtb*. As a rule, numerous extracellular growing bacteria were observed in an amorphous necrotic area at the luminal surface of the cavity, but only a few non-replicating pathogens were found in the caseous core of tuberculomas and small encapsulated granulomas [13,18,19,20]. Simultaneously, *Mtb*-infected host cells were rare in the tissue outside the necrotic mass of all types of the TB lung lesions examined [13,18,19,20,21,22]. These observations led to the view that *Mtb* is mainly an extracellular surviving and replicating pathogen, which spends a large portion of its lifespan in the caseous foci of TB patients’ granulomatous lesions [23]. The debate about the relative ratio and clinical relevance of extracellular and intracellular *Mtb* subpopulations, as well as about the tissue niches or cell types in which they reside in TB patients’ lungs, has been going on for decades [13,18,23,24]. At the same time, cellular proteomic, transcriptomic, and metabolomic data coming from the examination of TB patients’ lung injuries, basically, small fibrotic nodules and cavities, indicated that the inflammatory and immune responses of human cells differed substantially both between granulomatous lesions [10,18,19,20,21,22,25,26] and within the sub-compartments of granulomas [25]. In these studies, however, the protein and RNA expression profiles identified for the specific lung microenvironments were not investigated in relation to the *Mtb* load in host cells due to the inability to perform their adequate assessment.

We had previously developed a technique to produce ex vivo cell cultures, mainly of alveolar macrophages, from different tissues of lung parts surgically removed from patients with pulmonary TB, to determine the level of *Mtb* infection and the biological properties of both pathogen and human cells at the time of surgery [27,28,29]. Only single *Mtb* was detected in the alveolar macrophages of some TB patients, while the host cells of the other TB patients contained *Mtb* in colonies, including those with cording morphology, inside phagosomes [27,28,29]. The *Mtb* cords, when replicating the bacteria line up along their longitudinal axes in close parallel arrangement and setting themselves into “braids” or “ropes”, were associated with an increased virulence of *Mtb* in the guinea pig model of TB disease [28]. More *Mtb*-infected alveolar macrophages with increased numbers of *Mtb* in them were detected in the ex vivo cell cultures obtained from the cavity wall (approximately 40% of the cells examined) and the tissues distant from macroscopic TB lesions in the resected lungs of TB patients with cavities (from 1.88 to 6.54% of the host cells) [27]. In a special study [30], we indicated that the alveolar macrophages and other immune cells with *Mtb* in them, found in the ex vivo cell cultures, were isolated actually from TB patients’ lung tissues, and they characterize the TB infection in patients’ lungs at the time of surgery.

In this work, we compared the *Mtb* loads in the cells of the tuberculoma wall and, in parallel, in the lung tissue distant from the tuberculoma for each of eight patients with pulmonary TB disease and after intensive anti-TB chemotherapy before surgery. Then, we combined these data with an analysis of the activation and microbicidal statuses of alveolar macrophages and other cell subtypes examined both in the ex vivo cell cultures and on the histological sections obtained from the same TB patients’ local lung microenvironments. We detected unique molecular signatures of the immune, inflammatory, and hypoxia responses of cells for various lung lesions, with individual *Mtb* loads in them, for each TB patient. Our data revealed that bacterial control was associated with the inflammatory activation of cells and varying degrees of fibrosis in the discrete lesions in the lungs of the TB patients. Finally, the walls of tuberculomas with insufficient inflammation and excessive fibrosis were identified as being the main niche for *Mtb* survival (single or as colonies) in non-foamy alveolar macrophages among all the TB patients’ lung lesions examined.

## 2. Results

### 2.1. Histopathological Analysis Reveals Microenvironmental Features of the TB Patients’ Lung Lesions Used for Ex Vivo Expansion of Alveolar Macrophages

**Figure 1:**
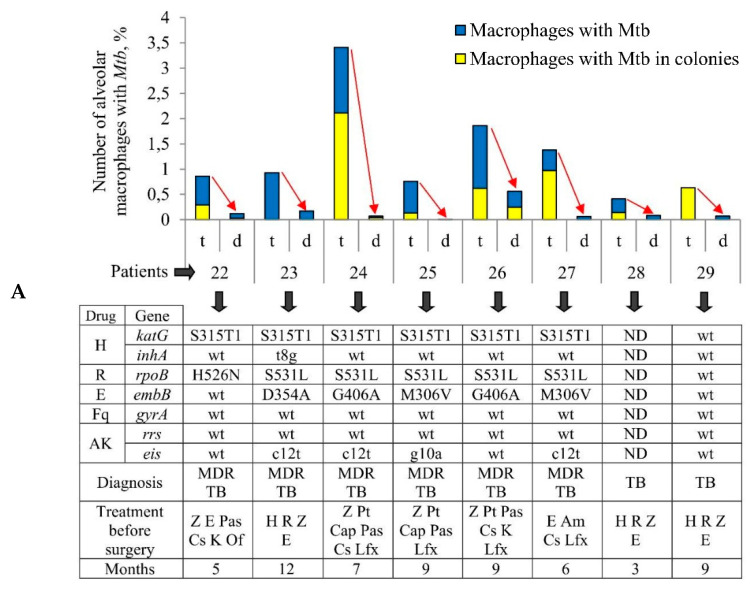

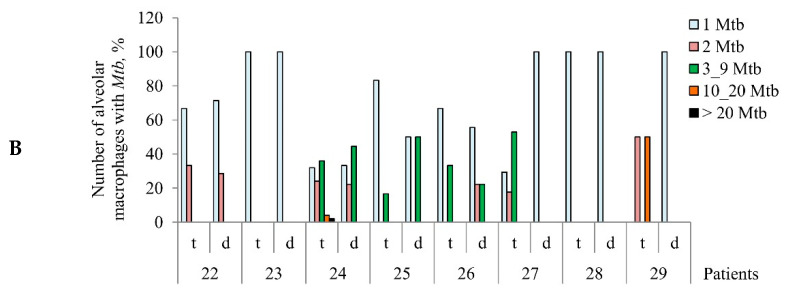
Increased numbers of alveolar macrophages with acid-fast *Mycobacterium tuberculosis* (*Mtb*) in them are observed in the ex vivo cell cultures obtained from tuberculoma walls (t), but not from lung tissues distant from tuberculomas (d) for the same tuberculosis (TB) patients. (**A**) The number of alveolar macrophages with any *Mtb* (single or as colonies) and with the colonies of *Mtb*, some with cording morphology, both expressed as the percentage of the total number of alveolar macrophages stained by the Ziehl-Neelsen method after ex vivo culture for 18 h. The table below the graph shows some characteristics of TB patients 22–29 (updated diagnosis after determination of drug resistance-associated mutations in the *Mtb* genes, treatment, and the months of therapy before surgery) and the bacteria (mutations in the *Mtb* genes associated with drug resistance), which infected the lungs of the TB patients. Drugs: AK, aminoglycosides; Am, amikacin; Cap, capreomycin; Cs, cycloserine; E, ethambutol; Fq, fluoroquinolones; H, isoniazid; K, kanamycin; Lfx, levofloxacin; Of, ofloxacin; Pas, para-aminosalicylic acid; Pt, protionamide; R, rifampicin; Z, pyrazinamide. ND, not determined; wt, wild type. Red arrows indicate a trend in the alteration of *Mtb* loads in alveolar macrophages between various lung lesions for the same TB patients. (**B**) The increased quantity of *Mtb* is detected in alveolar macrophages obtained from tuberculoma walls, but not from the lung tissue samples for the same TB patients. The number of alveolar macrophages with different numbers of *Mtb* expressed as the percentage of the total number of alveolar macrophages with *Mtb* after ex vivo culture for 18 h.

For ex vivo production of cells, we isolated the tuberculoma walls and the lung tissues distant from tuberculomas (“distant lung tissue samples” throughout) obtained from the surgically removed lung parts of eight patients (22–29) with pulmonary TB and after prolonged antibiotic therapy (Figure S1A,B). The anti-TB chemotherapy lasted 3 months for TB patient 28, 12 months for TB patient 23, and 5–9 months for the other TB patients (Figure 1A). For each TB patient, both specimens of lung tissues were rich in small necrotic nodules with fibrotic encapsulation up to 5 mm in diameter, which were separated in the sieves (Figure S1C) and then discarded. The cell suspension containing leukocytes was used to produce ex vivo cell cultures (Table 1, Figure S2A).

The histological examination of the Ziehl-Neelsen (ZN) and immunofluorescently stained sections of lung specimens revealed similar cellular and spatial architectures of the tuberculoma walls from the lungs of the TB patients studied. In the tuberculoma walls, a large central region of the necrotic caseum was surrounded by a thick dense fibrous rim containing numerous fibroblasts and abundant collagen with scattered alveolar macrophages, few multinucleated Langhans giant cells, some lymphocytes, very few pulmonary epithelial cells (also known as pneumocytes), and few big blood vessels among them (Table 1, Figures S2B and S3B–D).Lymphoid aggregates with several macrophages on the periphery of these areas were identified on the other side of the necrotic zone of the tuberculoma walls. Macrophages had a large number of denser dark inclusions in the cytoplasm and were what is called smoker’s cells for TB patients 27–29 (Table 1, Figure S2B). Few areas with the predominance of neutrophilic and macrophage infiltration together with the necrotic mass and debris of the dead cells were detected in some regions of the tuberculoma walls (Figure S3A). Single alveoli with few alveolar macrophages were observed in the tuberculoma walls of TB patients 27–29. Interestingly, this is the first time that a single megakaryocyte with a giant lobular nucleus was found in the tuberculoma wall (TB patient 28, Figure S3A), while one of our previous observations was that many megakaryocytes with the ex vivo monolayer cell cultures migrate from individual granulomas obtained from the spleens of mice after one month and two months of infection with the Bacillus Calmette–Guérin (BCG) vaccine [31].

For the lung tissues distant from tuberculomas, the examination of histological sections revealed a heterogeneous cellular architecture with diverse extents of pathology, including fibrosis and the preservation of the alveolar space. For TB patients 22 and 23, the cellular composition of the lung tissues distant from tuberculomas was similar to that observed for the tuberculoma walls. Accordingly, the absence of alveoli and extensive fibrosis with a mixed mononuclear-cell infiltrates consisting of few multinucleated Langhans giant cells and neutrophils, sheets of alveolar macrophages, and some scattered lymphocytes were found in these distant lung tissue samples (Table 1). Only minimal fibrosis in the interstitial area and multiple alveoli, which were filled with numerous smoker’s alveolar macrophages, few multinucleated Langhans giant cells, neutrophils, and lymphocytes, were observed in the distant lung tissue samples of TB patients 27–29 (Table 1, Figure S2B). Notably, some smoker’s alveolar macrophages were seen in the interstitial areas of the lung specimens of these patients, too. The distant lung tissue samples of TB patients 24–26 were characterized by focal remodeling of the interstitial zones with the proliferation of fibrous connective tissue in the large areas, but with a certain number of alveoli, which contained the clusters of alveolar macrophages, few multinucleated Langhans giant cells, neutrophils, and lymphocytes within the airspaces (Table 1, Figure S2B). No necrotic areas were identified in the distant lung tissue samples of the TB patients studied.

On the histological sections, single alveolar macrophages with acid-fast or immunofluorescently stained *Mtb* in them were detected both in the tuberculoma walls and in the alveolar space and interstitial regions of lung tissues distant from tuberculomas for the TB patients studied (Figures S2B, S3B and S4B). A large number of acid-fast *Mtb* was found only in the area of acellular caseous necrotic material, directly neighboring the cellular border of the tuberculoma wall for TB patient 26 (Table 1, Figure S2B). In contrast, in spite of a similar cellular and spatial architecture of the tuberculoma walls in the lungs of other TB patients, very few or no acid-fast *Mtb* were seen in the zones of the necrotic caseum (Table 1, Figure S2B). It is likely that the colonies of *Mtb* can enter the caseum from the dead cells with necrotic morphology in the tissues surrounding the caseum in the tuberculoma walls (Figure S3B). As we previously reported (see Table 1 in References [29,30]), all the TB patients studied were sputum smear negative and *Mtb* in the resected lung tissues of these patients were non-cultivable.

In general, the use of standard methods for identifying *Mtb* in human lung tissues did not allow us to evaluate the *Mtb* load in various lung lesions of the TB patients studied.

### 2.2. M. tuberculosis Loads in Alveolar Macrophages Vary between Different Lung Lesions for the Same TB Patients

To determine the exact number of *Mtb*-infected cells in various lung lesions, we isolated ex vivo cell cultures from the tuberculoma walls and, in parallel, the lung tissue distant from tuberculomas for each of the eight TB patients studied (Table 1, Figure S2A). At hour 18 of ex vivo culture, from 87% to 100% of the cells obtained from different lung lesions were found to be alveolar macrophages that were viable and had neither apoptotic nor necrotic morphology. Besides the alveolar macrophages, some viable dendritic cells, lymphocytes, and multinucleated Langhans giant cells were observed. Most of the alveolar macrophages obtained from both lung specimens of TB patients 27–29 had a large number of denser dark inclusions in the cytoplasm and were what is called smokers’ macrophages.

The number of acid-fast *Mtb*-infected alveolar macrophages varied significantly between the ex vivo cell cultures obtained from both the tuberculoma walls (from 0.41% for TB patient 28 to 3.41% for TB patient 24) and the lung tissues distant from tuberculomas (from 0% for TB patient 25 to 0.56% for TB patient 26) across the TB patients studied (Figure 1A). Only single *Mtb* were found in the alveolar macrophages of some TB patients, while *Mtb* in colonies were detected in the alveolar macrophages of other TB patients (Figure 1B, Figures S2A and S4E), some with cording morphology (for TB patients 24 and 26). However, for all TB patients studied, a significantly larger number of alveolar macrophages with *Mtb* in them were found in the ex vivo cultures of cells obtained from the tuberculoma walls, even though much fewer cells were isolated from these TB lesions compared to the lung tissues distant from tuberculomas (Table 1, Figure 1A). In addition, a larger number of alveolar macrophages contained two or more *Mtb* in colonies, including those with cording morphology, in the ex vivo cell cultures obtained from the tuberculoma walls of the TB patients (Figure 1A,B).

As described previously [29,30], *Mtb* from the resected lung tissues of TB patients 22–29 had similar characteristics, that is, they belonged to the Beijing genotype family and had low virulence in the guinea pig model of TB disease, but here the genetic analysis of several genes implicated in drug resistance revealed heterogeneity both in the presence/absence of resistance-associated mutations and in the number of these mutations in the DNA of *Mtb* among the TB patients studied regardless of the duration of anti-TB treatment before surgery (Figure 1A). Mutations for resistance to isoniazid, rifampicin, ethambutol, and aminoglycoside were identified in the *katG* and *inhA*, *rpoB*, *embB*, and *eis* genes, respectively, in the *Mtb* DNA for TB patients 22–27, with two to five mutations found. The resistance-associated genes were not analyzed for TB patient 28, probably due to a lack of *Mtb* DNA for analysis. For TB patient 29, the wild-type genes were present in *Mtb* DNA. 

Thus, despite the variation in the number of *Mtb*-infected alveolar macrophages between the ex vivo cell cultures examined, the same trend in the marked differences in bacterial burden existed among the lung specimens of all the TB patients studied: increased *Mtb* loads were detected in alveolar macrophages obtained from the tuberculoma walls, but not from the lung lesions distant from tuberculomas for the same TB patients, regardless of the distinct characteristics of both *Mtb* with substantial heterogeneity in the presence of drug resistance-associated mutations in their DNA between the TB patients’ lungs and the TB patients themselves after prolonged courses of intensive anti-TB therapy with various sets of antibiotics before surgery.

### 2.3. Transcription Factors HIF-1α and HIF-2α in Alveolar Macrophages Stabilize Differently across Various Lung Lesions and TB Patients

As is known [32,33,34], the HIF transcription factors, with two major oxygen-sensitive transcriptionally active HIF-1α or HIF-2α isoforms in them, are key elements in the control of metabolic and phenotypic reprogramming of myeloid cells to maximize the antimicrobial potential. In addition, they function as master regulators in cellular adaptation to hypoxia (low oxygen availability) in the lung lesions during TB infection associated with tissue dysfunction [10,11,12], which ultimately leads to tissue fibrosis. HIF-α isoforms are synthesized at a high rate but are rapidly targeted for ubiquitin-dependent proteasomal degradation. HIF-α stabilization in the cytoplasm of immune cells can be triggered by both hypoxia and other hypoxia-independent factors associated with inflammation, oxidative stress, and *Mtb* infection [10,11,12,32,33,34]. 

**Figure 2:**
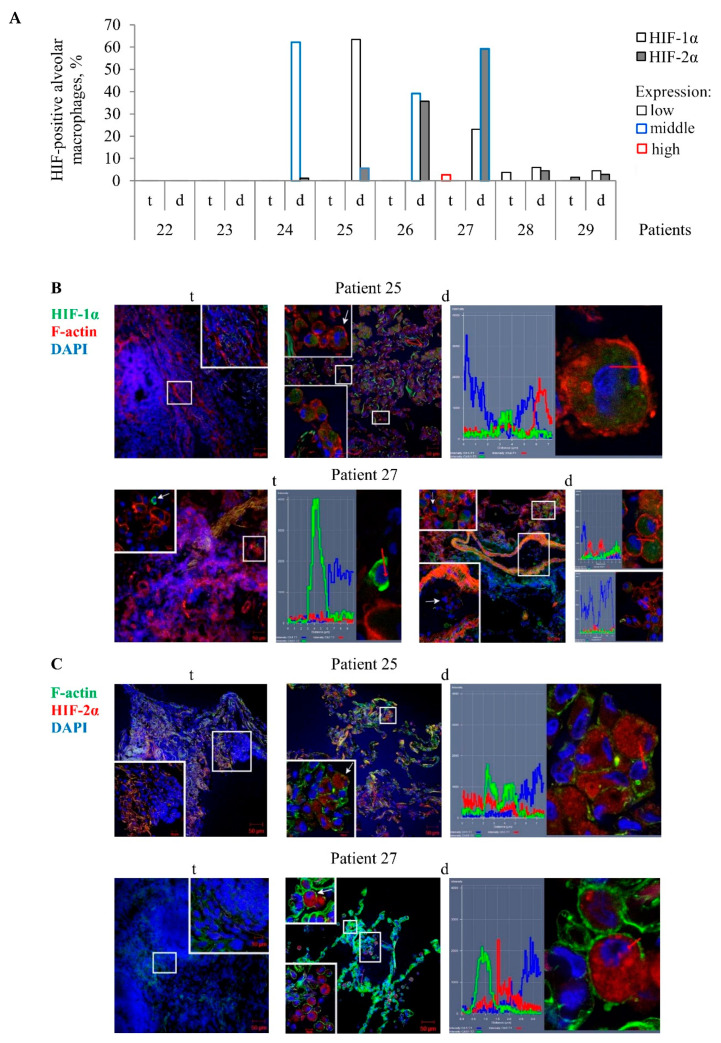
Alveolar macrophages with the transcription factors HIF-1α and HIF-2α accumulated in them are mainly detected on the histological sections obtained from the lung tissues distant from tuberculomas (d), but not from tuberculoma walls (t) for some TB patients. (**A**) The number of alveolar macrophages with the HIF-1α and HIF-2α isoforms expressed as the percentage of the total number of the TB patients’ alveolar macrophages analyzed. The expression of the markers is shown as low, middle, and high in black, blue, and red borderlines of bars, respectively. (**B**,**C**) Representative confocal fluorescent images of cells stained by antibodies to human (B) HIF-1α (green signal) or (**C**) HIF-2α (red signal) and phalloidin conjugated with (**B**) TRITC (red signal) or (**C**) Alexa 488 (green signal) show the location of these transcription factors in the cytoplasm, but not in the nuclei of alveolar macrophages. Nuclei are stained by DAPI (blue signal). Close-ups of the parts of these images with alveolar macrophages and other cells, with or without the examined markers in them, are shown in the corners. White arrows point to alveolar macrophages used to create the profile images. Red arrows identify the areas for the construction of profile images. The scale bars are 50 µm for the histological sections and 10 µm for the enlarged views of them.

In our work, the extent of fibrosis in the TB patients’ lung tissue samples showed considerable variation. For this reason, the study of the antibacterial activity of cells in various lung lesions of the TB patients was started with characterizing HIF-1α and HIF-2α protein expression by an immunofluorescent analysis of both lung specimens on the histological sections for each TB patient. To outline the cell contour and characterize the integrity of the plasma membrane of cells, the histological sections were simultaneously stained with phalloidin, which marks cortical actin filaments. The examination of different lung lesions revealed that numerous alveolar macrophages and multinucleated Langhans giant cells were immunoreactive for diffuse HIF-1α and HIF-2α staining in the cytoplasm, but not in the nuclei of alveolar macrophages mainly in the distant lung tissue samples of TB patients 24–27 (Figure 2A–C). Neither HIF-1α nor HIF-2α expression was found in the other cell subtypes, such as neutrophils, lymphocytes, fibroblasts, pneumocytes, or endothelial cells in blood vessels in both the tuberculoma wall and the lung tissue distant from the tuberculoma for each TB patient. In addition, the HIF-α isoforms were absent in the caseous necrotic mass in the tuberculoma walls. Interestingly, HIF-1α expression was determined in the alveolar macrophages of the alveolus, but not within monocytes in the nearby blood vessel on the histological section of TB patient 27 (Figure 2B). Notably, more alveolar macrophages with HIF-2α (59.3%) than with HIF-1α (23.16%) stabilization and with increased expression of HIF-2α in the cells were detected in the distant lung tissue sample of TB patient 27. In contrast, approximately identical percentages of HIF-1α- and HIF-2α-positive alveolar macrophages (39.22% and 35.57%, respectively) were observed in the distant lung tissue sample of TB patient 26, but the cells expressed more strongly HIF-1α than HIF-2α. In the tuberculoma wall of TB patient 27 (Figure 2A), the HIF-1α isoform was abundant in the cytoplasm of few alveolar macrophages with the absence of filamentous actin (Figure 2B), thus demonstrating HIF-1α stabilization in the cells with signs of necrotic death. In the tuberculoma walls of TB patients 28 and 29 (Figure 2A), some HIF-1α- and HIF-2α-positive alveolar macrophages, respectively, with weak filamentous actin staining were detected only in the areas with a large number of neutrophils and the accumulation of necrotic material (data not shown).

On the whole, the transcription factors HIF-1α and HIF-2α stabilization in the cytoplasm of alveolar macrophages varied across the lung lesions and the TB patients studied, with the HIF-1α isoform appearing to predominate in the cells of the distant lung tissue samples.

### 2.4. Transcription Factor NF-κB Subunits Are Expressed in Activated Alveolar Macrophages of the TB Patients’ Distant Lung Tissue Samples

*M. tuberculosis* is recognized by the PRRs, such as CD14, CD11b, and TLR-2, the latter two being expressed primarily on activated myeloid cells [6,7,8]. Binding of the PRRs activates downstream signaling through a central transcriptional regulator for innate immunity and inflammatory processes, the transcription factor NF-*κ*B consisting of p50 and p65 subunits, to control multiple aspects of immune cell functions [35].

**Figure 3:**
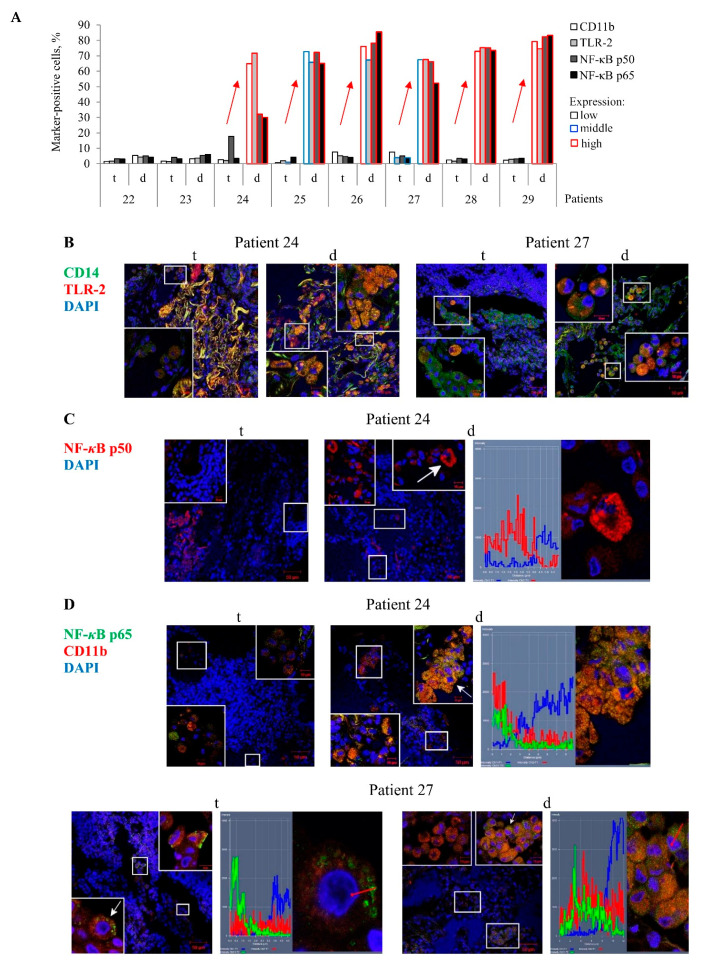
CD11b- and TLR-2-activated alveolar macrophages with an increased expression of the NF-*κ*B p50 and p65 subunits in them are detected on the histological sections obtained from the lung tissues distant from tuberculomas (d), but not from tuberculoma walls (t) for TB patients 24–29. (**A**) The number of alveolar macrophages with the markers expressed as the percentage of the total number of the TB patients’ alveolar macrophages analyzed. The expression of the markers is shown as in Figure 2A. Red arrows indicate a trend in the alteration of the number of the markers-positive alveolar macrophages between various lung lesions of the same TB patients. (**B**–**D**) Representative confocal fluorescent images of cells stained by antibodies to human (**B**) CD14 (green signal) and TLR-2 (red signal), (**C**) NF-*κ*B p50 (red signal), (**D**) NF-*κ*B p65 (green signal), and CD11b (red signal) demonstrate colocalization of some markers (yellow signal). Nuclei are stained by DAPI (blue signal). Close-ups of the parts of these images with alveolar macrophages and other cells, with or without the examined markers in them, are shown in the corners. The scale bars are 50 µm for the histological sections and 10 µm for the enlarged views of them. (**C**,**D**) The NF-*κ*B subunits localize in the cytoplasm, but not in the nuclei of alveolar macrophages. White arrows point to alveolar macrophages used to create the profile images. Red arrows identify the areas for the construction of profile images. (**B**, left panel) Collagen fibers are strongly autofluorescent in different wavelengths of emission spectra (yellow signal).

In an immunofluorescence assay of the histological sections, the PRRs TLR-2 and CD11b were found both on the cell surface, including in microdomains, and in large numbers of discrete granules in the cytoplasm of numerous markers-positive alveolar macrophages and multinucleated Langhans giant cells mainly in the distant lung tissue samples of TB patients 24–29, but not in the tuberculoma walls of the same TB patients (Figure 3A–C).For these TB patients, the NF-*κ*B p50 and p65 subunits were also detected in small roundish granules of varying size scattered peripherally to the nucleus, where the NF-*κ*B p65 subunit showed colocalization with the receptor CD11b, in a large number of the markers-positive alveolar macrophages, multinucleated Langhans giant cells, and some neutrophils in the lung tissues distant from the tuberculomas (Figure 3A,C,D). Approximately the same larger percentages of alveolar macrophages with increased expression of the PRRs TLR-2 and CD11b and NF-*κ*B subunits were normally observed only for the distant lung tissue samples of TB patients 24–29 (Figure 3A). In the tuberculoma walls of the TB patients studied, very few alveolar macrophages with the expression of these markers were found (Figure 3A–D). This phenomenon was also observable in the highly fibrotic areas of the distant lung tissue samples of TB patients 22 and 23. Only the receptor CD14 occurred at an increased density in both microdomains on the plasma membrane and the intracellular vesicles of most alveolar macrophages and multinucleated Langhans giant cells in all the TB patients’ lung lesions examined. In addition, the receptor CD14 often colocalized with the receptor TLR-2 in the markers-positive cells in the lung tissues distant from the tuberculomas (Figure 3B). Neither PRRs nor NF-*κ*B expression was observed in lymphocytes, fibroblasts, pneumocytes, or endothelial cells in blood vessels on the histological sections obtained from both lung specimens for each TB patient. In addition, no neutrophils and megakaryocyte with PRRs and NF-*κ*B expression were identified in the tuberculoma walls of the TB patients (Figure S3A). The expression of these markers was not found in the caseous necrotic mass of the tuberculoma walls for each TB patient. Interestingly, no accumulation of the transcription factor NF-*κ*B p50 and p65 subunits in the nuclei of alveolar macrophages was observed in the TB patients’ lung lesions examined (Figure 3C,D).

Thus, the enhanced expression of the NF-*κ*B subunits was predominantly found in the cytoplasm of activated alveolar macrophages mostly in the distant lung tissue samples of some TB patients.

### 2.5. The Antimicrobial Response of Alveolar Macrophages Correlates with Reduced Mtb Load in Them

During *Mtb* infection in human lungs, the activation of HIF and NF-*κ*B signaling pathways results in the release of many pro-inflammatory factors, including the enzymes inducible nitric oxide synthase (iNOS) and cyclooxygenase 2 (COX-2), which are responsible for the production of microbicidal nitric oxide (NO) and prostaglandin E2, respectively [11,25,33,35]. Prostaglandin production through the use of lipids compartmentalized in lipid droplets of foamy alveolar macrophages as well as ROS and NO generation are thought to be of major importance for regulating the antimicrobial action of myeloid cells in order to restrict the intracellular growth of *Mtb* and eliminate them [11,12,33].

**Figure 4:**
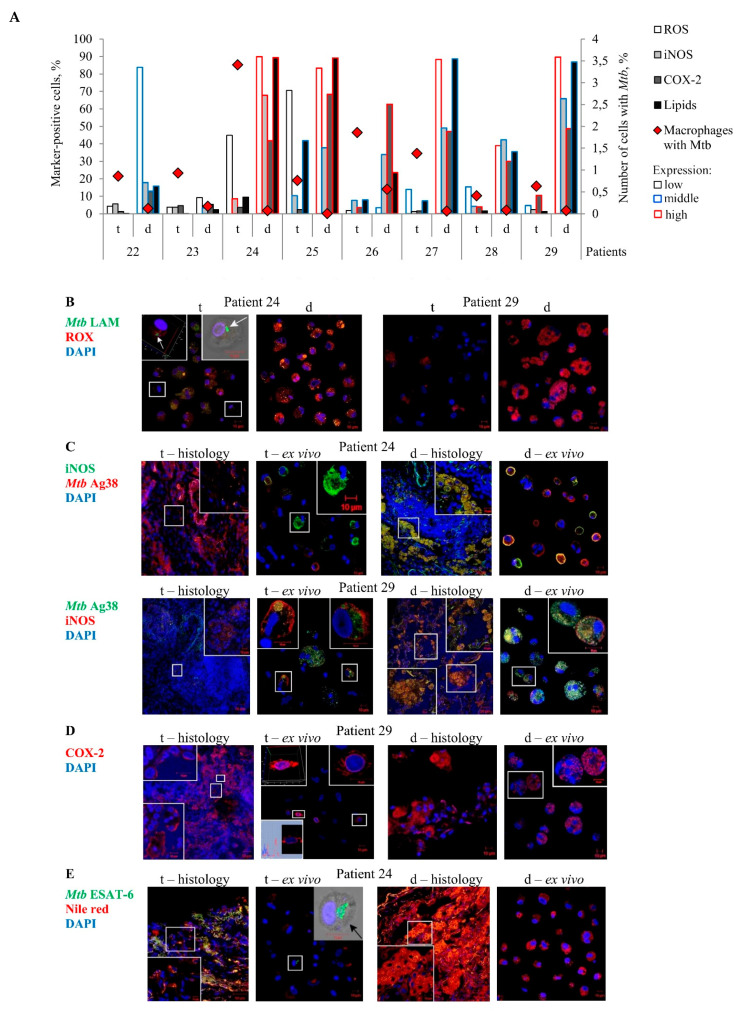
For the same TB patients, *Mtb*-infected alveolar macrophages are mainly found in the ex vivo cell cultures obtained from tuberculoma walls (t) with the lowest production of ROS, iNOS, COX-2, and lipids in very few cells, but not from the lung tissues distant from tuberculomas (d) with numerous foamy alveolar macrophages characterized by the bactericidal phenotype. (**A**) The number of alveolar macrophages with the markers expressed as the percentage of the total number of the TB patients’ alveolar macrophages analyzed after ex vivo culture for 18 h. The expression of the markers is shown as in Figure 2A. (**B**–**E**) Representative confocal fluorescent images of cells stained by antibodies to (**B**) *Mtb* lipoarabinomannan (LAM) (green signal), and the CellROX Deep Red Reagent (red signal), (**C**) human iNOS (green or red signals), and *Mtb* Ag38 (red or green signals), (**D**) human COX-2 (red signal), and (**E**) *Mtb* ESAT-6 (green signal) and the Nile red dye (red signal) demonstrate colocalization of some markers (yellow signal) on the histological sections (histology) and in the ex vivo cell cultures (ex vivo). Nuclei are stained by DAPI (blue signal). Close-ups of the parts of these images with alveolar macrophages and other cells, with or without the examined markers in them, are shown as 3D or phase contrasted confocal fluorescent images in the corners. The scale bars are 50 µm for the histological sections and 10 µm for the enlarged views of them and the images of the ex vivo cell cultures. (**B**) White and (**E**) black arrows point to *Mtb* cords in alveolar macrophages. (**D**, central-left panel) 3D and profile images of the same alveolar macrophage are shown in the left corners. (**C**,**D**) The expression patterns of iNOS and COX-2 differ between the histological sections and the ex vivo cell cultures obtained from the same lung specimens.

In our study, ROS biosynthesis was examined only in alveolar macrophages analyzed after ex vivo culture for 18 h, while the estimation of lipid content and the immunofluorescencent visualization of iNOS and COX-2 expression in the cells were performed both on the histological sections and in the ex vivo cell cultures obtained from the same lung lesions for each TB patient (Figure 4A–E). Oxygen radicals were predominantly found in the intracellular vesicles of the marker-positive alveolar macrophages and multinucleated Langhans giant cells (Figure 4B). Accumulation of lipids in the marker-positive alveolar macrophages and multinucleated Langhans giant cells was observed in cytoplasmic granules both on the histological sections and in the ex vivo cell cultures obtained from the same lung specimens of the TB patients (Figure 4E). However, characteristic staining patterns associated with the enzymes iNOS and COX-2 expression in the markers-positive TB patients’ cells differed between the histological sections and the ex vivo cell cultures obtained from the same lung specimens. On the histological sections, these compounds were detected only in vesicles of different size in the cytoplasm of the markers-positive alveolar macrophages and multinucleated Langhans giant cells (Figure 4C,D). In the ex vivo cell cultures, the majority of iNOS expression patterns were associated with the plasma membrane of the marker-positive alveolar macrophages obtained from both the tuberculoma walls and the distant lung tissue samples of the TB patients (Figure 4C). It is possible that the iNOS molecules were actively secreted by these cells in ex vivo culture. Some alveolar macrophages in the ex vivo cell cultures obtained from the tuberculoma walls of TB patients 28 and 29 demonstrated the COX-2 expression in the areas corresponding to the location of endoplasmic reticulum (Figure 4D),while in other marker-positive cells, COX-2 molecules were defined in intracellular vesicles (Figure 4D). No expression of the markers examined was detected in neutrophils, lymphocytes, fibroblasts, or endothelial cells in blood vessels on the histological sections obtained from both the tuberculoma walls and the distant lung tissue samples of the TB patients (Figure 4C–E and Figure S3C,D). For some TB patients, very few pneumocytes produced a small number of COX-2 molecules and sometimes lipids.

In the tuberculoma walls, the lipids, including lipid droplets of different size, were abundant only in the necrotic caseum and dead necrotic cells, including alveolar macrophages and fibroblasts located either inside or immediately adjacent to the necrotic mass. The lipids were accumulated both extracellularly and intracellularly in the dead cells on the histological sections of all TB patients (Figure S3C). These areas were also characterized by iNOS, but not COX-2, expression and the presence of the *Mtb* antigens: 6-kDa early secretory antigenic target (ESAT-6) and Ag38 (Figure S3C,D)—although intact visible bacteria were generally absent in the TB patients’ cells. In addition, only single *Mtb* were found in the necrotic zones of the tuberculomas for the majority of the TB patients studied (Table 1, Figures S2B and S3B–D). In addition to the necrotic morphology of cells with the plasma membrane broken down and some nuclei destroyed, further evidence that the cells labeled intensely with the markers in the regions surrounding the caseum of the tuberculoma walls were dead was provided by the fact that such markers-positive cells were not detected in the ex vivo cell cultures obtained from the same lung tissue parts, since the dead cells could not attach to the cover glasses after ex vivo expansion from the resected lungs and were removed with cell debris after ex vivo culture for 18 h. Notably, the markers and *Mtb* antigens examined were often revealed together in both the necrotic caseum of the tuberculoma walls and intracellular vesicles of the markers-positive alveolar macrophages and multinucleated Langhans giant cells on the histological sections and cell preparations obtained from various lung lesions of the TB patients (Figure 4B,C,E and Figure S3C,D). 

Despite the distinct characteristics of the expression patterns, the number of markers-positive alveolar macrophages was generally the same both on the histological sections and in the ex vivo cell cultures for each TB patient (Figure 4C–E). Thus, the exact number of alveolar macrophages with the iNOS and COX-2 expression, lipid production as well as ROS generation in them was evaluated in the ex vivo cultures of cells (Figure 4A). Substantial variations in the number of alveolar macrophages with the expression of the corresponding markers were observed among the TB patients studied. Small numbers of markers-positive alveolar macrophages were normally found in the ex vivo cell cultures obtained from the tuberculoma walls of all TB patients and the distant lung tissue samples of TB patients 22 and 23. In comparison with the tuberculoma walls, the ex vivo cell cultures obtained from the distant lung tissue samples of TB patients 24–29, where there were very few *Mtb*-infected alveolar macrophages, displayed a higher number of alveolar macrophages with increased ROS generation and iNOS, COX-2, and lipid production (Figure 4A). Noticeably, distant lung tissue samples with numerous pro-inflammatory and antimicrobial factors-producing alveolar macrophages in the resected lungs of some TB patients also contained host cells with *Mtb* in colonies, including those with cording morphology (Figure 1A,B, Figures S2A,B and S4A–E). The colonies of *Mtb* with cording morphology in alveolar macrophages obtained from the distant lung tissue samples were similar to colonies of *Mtb* in some host cells obtained from the tuberculoma walls for the same TB patients (Figure S4E). In addition, *Mtb* was found both in the markers-positive and in the markers-negative alveolar macrophages in the ex vivo cell cultures obtained from the distant lung tissue samples of TB patients 24–29 (Figure S4A–D). Note that the use of different staining techniques (ZN and Ag38/ESAT-6/LAM immunofluorescent staining) produced similar numbers of both *Mtb*-infected alveolar macrophages and *Mtb* in them for all the histological and cytological preparations analyzed for each TB patient.

On the whole, a higher *Mtb* load in alveolar macrophages was associated with reduced pro-inflammatory and bactericidal activity of cells in the tuberculoma walls and, by contrast, only very few alveolar macrophages with *Mtb* in them were found in the lung tissues distant from tuberculomas, where the cells mainly demonstrated an increased microbicidal potential, for the same TB patients.

### 2.6. Tuberculoma Walls Are the Main Niche for Mtb Survival in Host Cells among Various TB Patients’ Lung Lesions Examined

High levels of pro-inflammatory and antimicrobial factors that are released by activated alveolar macrophages during TB infection in human lungs promote the killing of the pathogen, but may lead to tissue damage and abnormal repair of lung lesions resulting in tissue fibrosis. Based on an anatomical assessment of the TB patients’ lung lesions, the lung specimens examined were divided into several groups. The “tuberculoma” group included the lung tissue samples obtained from the tuberculoma walls of all TB patients studied (*n* = 8). On the histological sections, these macroscopic TB lesions were uniformly characterized by a big mass of the necrotic caseum at the central zones and excessive fibrosis in the tissues surrounding the caseum. The “distant part” group included the lung tissue samples obtained from the lung parts distant from macroscopic TB lesions, including the tuberculomas, in all TB patients studied (*n* = 8). However, the histological and morphological examination of the distant lung tissue samples showed considerable variation in the extent of fibrosis among the TB patients studied. Consequently, the distant lung tissue samples of TB patients 22 and 23 with extensive fibrosis and the absence of alveoli were further assigned to the “type I” group. The distant lung tissue samples of TB patients 24, 25, and 26 were found to have only focal fibrosis and some alveoli. Consequently, they were assigned to the “type II” group. The “type III” group included the rest of the distant lung tissue samples of TB patients 27, 28, and 29 with minimal signs of fibrosis and lots of alveoli.

**Figure 5:**
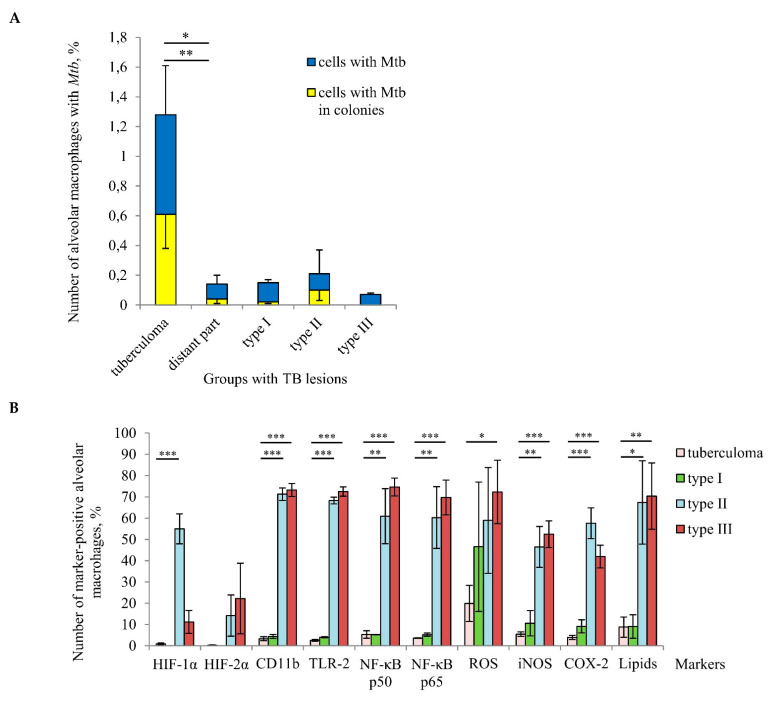
A tuberculoma wall with the lack of antimicrobial activity of alveolar macrophages is the main niche for *Mtb* survival (single or as colonies) in host cells among different TB patients’ lung lesions examined. (**A**) The number of alveolar macrophages with any *Mtb* (single or as colonies) and with *Mtb* in colonies, some with cording morphology, both expressed as the percentage of the total number of alveolar macrophages. (**B**) The number of alveolar macrophages with the markers expressed as the percentage of the total number of the TB patients’ alveolar macrophages analyzed. (**A**,**B**) The TB lesions examined are the tuberculoma walls (“tuberculoma” group) and the lung tissues distant from tuberculomas (“distant part” group) of all TB patients studied, and, differentially, the lung tissues with extensive, focal, and minimal fibrosis (“type I”, “type II”, and “type III” groups, respectively) distant from tuberculomas of TB patients 22–23, 24–26, and 27–29, respectively. Data are expressed as the means ± SEM. (**A**) * *p* ˂ 0.05 (comparing alveolar macrophages with *Mtb* in colonies), ** *p* < 0.01 (comparing alveolar macrophages with *Mtb*), Student’s *t*-test. (**B**) * *p* ˂ 0.05, ** *p* < 0.01, *** *p* < 0.001, Student’s *t*-test.

A comparison of infection parameters for various TB patients’ lung lesions demonstrated that a considerable increase in the number of alveolar macrophages with *Mtb* (single or as colonies) occurred in the “tuberculoma” group compared to the other groups, which included distant lung tissue samples (Figure 5A). Notably, the different groups made up of distant lung tissue samples had different morphological characteristics, but displayed approximately identical *Mtb* loads in host cells. At the same time, a very low number of alveolar macrophages with iNOS and COX-2 expression, ROS generation, and lipid production were found in the “tuberculoma” group (Figure 5B). Moreover, the expression of the activation markers, such as the PRRs CD11b and TLR-2, and the NF-*κ*B p50 and p65 subunits, was very low in alveolar macrophages from the “tuberculoma” group compared to that in the “type II” and “type III” groups, where a substantial production of the markers examined was demonstrated in alveolar macrophages (Figure 5B). Only alveolar macrophages from the “type I” group had approximately the same characteristics of the markers examined as the cells from the “tuberculoma” group.

HIF-1α and HIF-2α stabilization in the TB patients’ cells was virtually unobserved in the “tuberculoma” group and was absent from the “type I” group, while the lung tissue samples in both groups were characterized by an increased level of tissue fibrosis and, probably, alveolar macrophages in such pathologically fibrotic tissues were under hypoxic conditions. Interestingly, the expression of HIF-α isoforms in alveolar macrophages from the “type II” and “type III” groups showed inverse dynamics: the number of cells with HIF-1α stabilization was increased in the “type II” group as compared with the “type III” group. However, a higher number of cells with HIF-2α expression was detected in the “type III” group (Figure 5B).

Taken together, our results demonstrated that tuberculoma walls with insufficient inflammation and excessive fibrosis were the main niche for *Mtb* survival (single or as colonies) in alveolar macrophages among various lung lesions examined. At the same time, the distant lung tissue samples with both the reduced and increased pro-inflammatory activity and microbicidal potential of alveolar macrophages located in extensive or minimal/focal fibrotic tissues, respectively, were characterized by low *Mtb* loads in host cells for all the TB patients studied.

## 3. Discussion

Despite half a century of anti-TB chemotherapy, pulmonary TB is still one of the world’s major health problems, caused by the pathogenic mycobacteria with the formation of a broad range of abnormal lung lesions within a single host [1,5]. In our study, we analyzed *Mtb* loads in human cells and the activation and bactericidal statuses of alveolar macrophages in the tissue specimens and the ex vivo cell cultures obtained from discrete lung TB lesion types, such as macroscopic lung injuries classified as tuberculomas according to the Russian National Guidelines for Phthisiology [16], and the lung tissue distant from tuberculomas for each of the eight patients with pulmonary TB disease and after intensive anti-TB chemotherapy before surgery. In our work, the tuberculomas examined had morphological signs of progressive TB lesions and were not identical to the tuberculomas that were obtained from patients undergoing surgery for reasons unrelated to TB disease and characterized as “nonprogressive lesions” in a study by Ulrichs et al. [21].

Our data identify the tuberculoma walls as the main niche for *Mtb* survival (single or as colonies) in alveolar macrophages among various lung lesions examined. We disagree with Marakalala et al. [25] and believe that live cells, including alveolar macrophages with or without *Mtb* in them, located in all areas of the TB patients’ tuberculoma walls, in particular in the tissue immediately adjacent to the necrotic caseum, are characterized mainly by an anti-inflammatory signature with the absence of both lipid production and the biosynthesis of bactericidal compounds, such as oxygen and nitrogen radicals. A small number of TB patients and lung TB lesions examined were a limitation in our work. However, we obtained the same results for all TB patients studied, while they were characterized by both distinct courses of intensive anti-TB therapy with various sets of antibiotics before surgery and different *Mtb* with significant heterogeneity in the presence of drug resistance-associated mutations in the lung tissues between the TB patients. In our earlier work [27], we tested alveolar macrophages obtained from two lung tissue samples of TB patient 12, collected simultaneously from the lung tissues at 5 cm away from one side of the tuberculoma and 12 cm away from the other side, and found approximately the same characteristics of the *Mtb* load in host cells and the bactericidal and inflammatory activity of alveolar macrophages in ex vivo culture (data not shown). As a result of that preliminary study, here we used substantially more morphologically different TB lung lesions for analysis.

Remarkably, the lung tissues distant from the tuberculomas of the majority of the TB patients studied were characterized by the presence of multiple alveolar macrophages with strong pro-inflammatory activation and an increased microbicidal potential and a very low number of *Mtb*-infected cells. However, these distant lung tissue samples still contained both pro-inflammatory activated and non-activated host cells with single *Mtb* or *Mtb* in colonies, including those with cording morphology. We hypothesize that alveolar macrophages can migrate not only from the alveolar space to the tuberculoma wall, as smoker’s alveolar macrophages, but also in the opposite direction, including migration of *Mtb*-infected cells into inflammatory tissues distant from tuberculomas, probably, due to a gradual increase in chemokine gradient or the HIF-1α-dependent accelerated migratory capacity of macrophages, as recently observed in a study by Semba et al. [36]. In our earlier work [27], an increased number of *Mtb*-infected alveolar macrophages in the distant lung tissue samples of TB patients correlated, first of all, with the presence of cavities, the walls of which were observed to have a substantial *Mtb*. Thus, not only cavities, where *Mtb* replication is enhanced due to improved access to oxygen, promote *Mtb* dissemination both outside the organism with sputa coughed out and within the lungs, but also tuberculomas with a much smaller number of *Mtb*-infected cells in their walls serve as niches for *Mtb* survival in the TB patients’ lungs and, perhaps, the spread of TB infection to other lung tissues of TB patients. These assumptions, however, require further studies.

It is important to note that the high pro-inflammatory and antimicrobial activation of alveolar macrophages in the distant lung tissue samples of some TB patients was probably the result of the continued persistence of a large number of intracellular vesicles with *Mtb* products in them. In other studies [37,38], different *Mtb* antigens were also detected in many human alveolar macrophages on histological sections from the resected lungs of TB patients, where *Mtb*-infected cells were rare. As reviewed in [39,40,41], *Mtb* products that have been stored in the vesicular compartments for a long time can modulate (directly or indirectly) the immune response to best protect the host. However, if these vesicles promote the induction of an excessive immune response, the inflammatory mediators and bactericidal compounds can persist in the lung tissues long after *Mtb* clearance and induce lung tissue damage, which ultimately leads to dysfunctional alveolar remodeling and tissue fibrosis. Probably, this process was observed in the distant lung tissue samples of the TB patients studied.

Although an increased expression of the NF-*κ*B subunits in the cytoplasm of alveolar macrophages is correlated with high rates of ROS biosynthesis, TLR-2, iNOS, and COX-2 production in them, we did not observe their accumulation in the nuclei of activated cells. It was shown that the pathogenic *M. avium*, unlike the nonpathogenic mycobacteria, represses NF-*κ*B activation for survival within the cells of mouse monocyte-like cell lines [9]. On the other hand, as reviewed in [42], NF-*κ*B signaling in various human myeloid cells stimulated in vitro with different ligands can be encoded by the period and amplitude of NF-*κ*B nuclear/cytosol oscillations by which NF-*κ*B proteins shuttle between the cytoplasm and the nucleus to initiate transcription of different genes. Whether the dynamics of NF-*κ*B translocation to the nucleus of alveolar macrophages are involved in regulating the quality and quantity of the inflammatory response in the lungs of TB patients remains to be known.

The HIF-1α and HIF-2α stabilization in the cytoplasm of cells is usually associated with different polarization state of macrophages, when HIF-1α drives the expression of pro-inflammatory molecules and release of NO, whereas HIF-2α predominates in the reparative stage of inflammation in anti-inflammatory macrophages [32,33,34,43]. In our work, the stabilization of the HIF-α isoforms in the TB patients’ alveolar macrophages was not directly correlated with cellular inflammatory and antimicrobial activation or the degrees of fibrosis. We detected varying levels of accumulations of both isoforms in the cytoplasm of alveolar macrophages, but not in the nuclei of cells, throughout the lung lesions and the TB patients studied. How these HIF-α isoforms contribute to antimicrobial control and help alveolar macrophages to adapt to the microenvironmental changes associated with inflammation and tissue damage in various TB patients’ lung lesions is the matter of further research.

Foamy alveolar macrophages in the lungs of TB patients are thought to provide lipid-rich nutrients, which are used by *Mtb* for energy synthesis, virulence factor expression, cell wall and outer membrane construction [11,44]. Nonetheless, both here and in our earlier work [27,29], we revealed replicating *Mtb* in colonies, including those with cording morphology, in alveolar macrophages without lipid droplets in them. Our data suggest that lipid availability is primarily important for inflammatory and antimicrobial activated alveolar macrophages as fatty acids and cholesterol support the production of pro-inflammatory molecules and maintain homeostasis in the TB patients’ cells in hypoxic and inflamed hostile microenvironments. Thus, lipid-rich alveolar macrophages in the lungs of TB patients must be considered, above all, as structural markers of inflammation, but not as a niche with a nutrient source for *Mtb* survival and replication within cells.

Our results suggest that the tuberculoma walls of all TB patients studied and the distant lung tissue samples of some TB patients had simultaneously increased levels of fibrosis and reduced pro-inflammatory activity and microbicidal potential of alveolar macrophages located in these lung lesions. These data agree well with the assumption that fibrosis may provide protection against excessive inflammation and to be an inflammation outcome. At the same time, higher *Mtb* loads in alveolar macrophages were found in the tuberculoma walls. Thus, reduced inflammation and defective antimicrobial activity result in intracellular survival of *Mtb* (single or as colonies) depending on the TB lesion type.

While we revealed differences in the localization of some markers between alveolar macrophages on the histological sections and after ex vivo culture for 18 h both here and in our earlier work [30], the number of markers-positive alveolar macrophages detected by different testing methods was generally the same for each TB patient studied. Therefore, our technique to produce ex vivo cultures of cells, mainly of alveolar macrophages, from different lung tissues allows us to assess not only the level of *Mtb* infection and the functional status of the pathogen, but also the production of inflammatory and bactericidal compounds in various TB patients’ lung lesions in an unprecedentedly short time. The proposed method is very important to evaluate and compare the infection statuses relative to the immune response of host cells, especially in the distant lung tissue samples where the characteristics of both *Mtb* and human cells revealed here and in our earlier work [27,29] varied greatly across the TB patients. These data are necessary to develop and revise individual strategies in post-operative treatment of patients with pulmonary TB within the concepts of personalized medicine and adjunctive host-directed therapies that should be aimed at creating a balance between mounting an effective immune reaction against *Mtb* and protecting lung tissues from hyperinflammation.

## 4. Materials and Methods

### 4.1. Patients and Lung Tissue Samples

Lung tissue samples were obtained from patients 22–29 with pulmonary TB at the Department of Thoracic Surgery of the Ural Research Institute for Phthisiopulmonology (Yekaterinburg, Russia) affiliated with the National Medical Research Center of Tuberculosis and Infectious Diseases of the Ministry of Health of the Russian Federation (Moscow, Russia) as described in [27]. The patient nomenclature used is explained in [27]. These patients had previously been characterized in detail (age, treatment, attendant diseases, surgery, and others) (see Table 1 in [29,30]). In brief, all patients had tuberculomas and other fibrotic and caseotic TB lesions in the lungs and had been referred for the surgical management of pulmonary TB. All procedures involving patients were fully reviewed and approved by the Ethical Committee of the Ural Research Institute for Phthisiopulmonology (27/2014/07/02) and conducted in accordance with the principles expressed in the Helsinki Declaration. Written informed consent was obtained from each patient in this study. For each TB patient, immediately after surgery, samples of lung tissue (10–15 g) were collected simultaneously from the tuberculoma wall and from the lung tissue about 5 cm away from the tuberculoma. 

### 4.2. Ex Vivo Production of TB Patients’ Alveolar Macrophages

Alveolar macrophages from the samples of surgically resected lung tissue of the patients were produced as described in [27]. In brief, samples of lung tissue were cut into small pieces and rubbed through a metal screen of a sieve with pores 0.5–2.0 mm in diameter in phosphate-buffer saline (PBS, pH 7.4) for separating the cell suspension containing alveolar macrophages from closed granulomatous-fibrotic tissue. Cell pellets were centrifuged at 400 *g* for 5 min at room temperature and placed to 24-well plates (Orange Scientific, Belgium) with cover glasses (approximately 8 × 8 mm in size) in the bottom and cultured for 18 h in 0.5 mL of RPMI 1640 complete growth medium containing 10% fetal bovine serum, 2 mM glutamine, and 50 µg/mL gentamicin (BioloT, Russia) at +37 °C in an atmosphere containing 5% CO_2_.

### 4.3. Analysis of Drug-Resistance Mutations in Mtb Genes

For isolation of mycobacterial DNA, the lung tissue samples obtained from the surgically resected lung parts of TB patients were incubated for 60 min in 0.5 mL of solution for *Mtb* inactivation (Sintol, Moscow, Russia), then an Amplitube kit (Sintol, Moscow, Russia) was used for DNA extraction. Mutations associated with resistance to isoniazid (*katG*, *inhA*), rifampicin (*rpoB*), ethambutol (*embB*), fluoroquinolones (*gyrA*, *gyrB*), capreomycin and aminoglycosides (*rrs*, *eis*), and the *Mtb* genotype (Beijing, Beijing B0, Haarlem, LAM, Ural) were analyzed using a microarray TB-TEST assay (BIOCHIP-IMB, Moscow, Russia) according to the manufacturer’s instructions.

### 4.4. Cell Staining

At hour 18 of ex vivo culture, the TB patients’ cell cultures on cover glasses were washed with PBS and fixed with 4% formaldehyde solution in PBS for 10 min at room temperature. To visualize acid-fast *Mtb* within host cells, some of the patients’ fixed cell preparations were washed with PBS and stained by the ZN method. After ZN staining, the cells were counterstained with Mayer’s hematoxylin.

In an immunofluorescence assay with using dyes and/or antibodies, the cell preparations were incubated with 10 µM of Nile red dye (Invitrogen, USA, N1142) or 5 µM of ROS detection CellROX Deep Red Reagent (Molecular Probes, USA, C10422) for 15 or 30 min, respectively, at +37 °C in 5% CO_2_ before fixation. Next, the cell preparations were fixed as described above, washed with PBS, blocked in PBS solution containing 2% BSA (but not for CD markers staining), and finally, incubated first with primary antibodies, then with secondary antibodies. Primary antibodies were against *M. tuberculosis* Ag38 (Abcam, England, ab183165, 1:1000 dilution), ESAT-6 (courtesy of E.V. Deineko, Federal Research Center Institute of Cytology and Genetics, SB RAS, Novosibirsk, Russia, 1:300 dilution), LAM (Abcam, England, ab20832, 1:200 dilution), and human CD14 (Spring Bioscience, USA, M492, diluted 1:100 dilution), COX-2 (Santa Cruz, USA, sc-1747-R, 1:50 dilution), iNOS (Spring Bioscience, USA, E374, 1:100 dilution), TLR-2 (BioLegend, USA, 309708, 1:200 dilution). Fluorescent visualization of markers was enabled using secondary goat polyclonal Alexa 488-, DyLight 488-, and DyLight 594-conjugated anti-rabbit IgG (Thermo Fisher Scientific, USA, A11034, 35553, and 35561, respectively, 1:400 dilution), Alexa 488-conjugated anti-mouse IgG (Thermo Fisher Scientific, USA, A11001, 1:400 dilution), Alexa 555-conjugated anti-mouse IgG (Invitrogen, USA, A21422, 1:500 dilution) antibodies. The cell preparations were incubated with the appropriate antibodies for 60 min at room temperature. Fluorescent staining was analyzed using the VECTASHIELD Mounting Medium with DAPI (4′,6-diamidino-2-phenylindole) (Vector Laboratories, USA, H-1200).

### 4.5. Histology

The histological sections of the resected lung tissues of the TB patients were prepared as described in [29]. In brief, the resected lung parts of the TB patients (the tuberculoma wall and the lung tissue distant from the tuberculoma) were cut into pieces. One portion of lung pieces was collected for producing alveolar macrophages as described above. The other portion of lung pieces was fixed with 4% formaldehyde solution in PBS (pH 7.4) for 20 h at +4 °C. After fixation, the lung tissues that had no small encapsulated granulomas in them were washed with PBS, incubated with 30% sucrose in PBS (pH 7.4) for 20 h at +4 °C, frozen in Tissue-Tek O.C.T. Compound (Sakura Finetek, USA, 4583) at −25 °C, and sectioned at 16-µm slides on a Microtome Cryostat HM550 (Microm, Germany) at the Shared Center for Microscopic Analysis of Biological Objects of the Institute of Cytology and Genetics, SB RAS (Novosibirsk, Russia). Sections were air-dried on SuperFrost Plus slides (Thermo Fisher Scientific, USA) and stained by the ZN method. 

The histological sections were treated within 45 min in 0.3% Triton-X100 solution, blocked, and immunofluorescently stained with antibodies described above. Additional primary antibodies were against human CD11b (EBioscience, USA, 17-0112-81, 1:200 dilution), HIF-1α and HIF-2α (Sigma, USA, AB2702132 and AB2701992, respectively, 1:500 dilution), NF-*κ*B p50 (BioVision, USA, 3354, 1:200 dilution) and p65 (BioVision, USA, 3038, 1:50 dilution). Alexa 488-labeled phalloidin (Invitrogen, USA, A12379, 1:100 dilution) and TRITC-labeled phalloidin (Sigma, USA, P1951, 1:100 dilution) dyes were used to stain filamentous actin.

All the histological sections were incubated with the appropriate primary antibodies for 20 h at +4 °C and with the appropriate secondary antibodies for 60 min at room temperature. Fluorescent staining was analyzed using the ProLong Gold Antifade Mountant with DAPI (Thermo Fisher Scientific, USA, P36935).

### 4.6. Microscopy

The histological sections and the cytological preparations were examined at the Shared Center for Microscopic Analysis of Biological Objects of the Federal Research Center Institute of Cytology and Genetics, SB RAS (Novosibirsk, Russia), using an Axioskop 2 plus microscope (Zeiss) and objectives with various magnifications (Zeiss), and photographed using an AxioCam HRc camera (Zeiss); the images were analyzed using the AxioVision 4.7 microscopy software (Zeiss). The preparations stained with fluorescent dyes and antibodies were examined under an LSM 780 laser scanning confocal microscope (Zeiss) using the LSM Image Browser and ZEN 2010 software (Zeiss).

The human cells and *Mtb* in host cells on the cytological preparations were counted separately on each cover glass for each TB patient. More than 1000 alveolar macrophages were analyzed at each preparation for each TB patient. 

For the histological preparations, two un-serial tissue sections from each individual sample and five areas per each tissue section were analyzed for cell composition, for the markers by immunofluorescent staining of caseum and for each cell type. A semi-quantitative analysis was chosen to estimate the number of cells on the histological preparations: the (+++), (++), (+), and (-) indicators were used to describe the tissue sections where approximately 30–50%, 10–20%, 0.1–5%, and 0% of cells, respectively, belonged to a particular cell type. 

On the graphs, the expression of markers in alveolar macrophages was shown as low, middle, and high according to a two-, a three- and a four-fold increase in the intensity of the immunofluorescent staining of the marker in alveolar macrophages as compared to the background staining of lung epithelial cells and fibroblasts on the confocal profile, respectively.

### 4.7. Statistical Analysis

Statistical data processing was performed using Microsoft Excel 2010 and GraphPad Prism 6.0 (GraphPad Software). Statistical significance for the comparisons between the datasets was determined using Student’s *t*-test. Differences were considered statistically significant at *p* < 0.05.

## 5. Conclusions

In the present study, we identified unique molecular signatures of the immune, inflammatory, and hypoxia responses of cells for various lung lesions, with individual *Mtb* loads in them, obtained from the resected lungs of patients with pulmonary TB and after intensive anti-TB chemotherapy before surgery. Our data revealed that bacterial control was associated with the inflammatory activation of alveolar macrophages and varying degrees of fibrosis in the discrete TB lung lesions. Tuberculoma walls with insufficient inflammation and excessive fibrosis were identified as the main niche for *Mtb* survival (single or as colonies) in non-foamy alveolar macrophages among the TB patients’ lung lesions examined. The identification of immune, inflammatory, and bactericidal factors relative to estimating the infection status of alveolar macrophages engaged in the pathological process within various lung lesions will allow design of therapies directed toward modulating the immune response to best protect the host, controlling and ultimately stopping the pathology, and arresting transmission of *Mtb* both outside and throughout lungs of TB patients.

## Figures and Tables

**Table 1:** Cells on histological sections and in ex vivo cell cultures (^1^) obtained from the tuberculoma walls (t) and the lung tissues distant from tuberculomas (d) for the same TB patients.

Patient No.	Lung Tissue	*Mycobacterium tuberculosis (Mtb*) in Caseum ^2^	Pneumocytes	Macrophages	Fibroblasts	Lymphocytes	Neutrophils	Lang-hans Giant Cells	Dendritic Cells	Mega-karyocytes	Total Number of Ex Vivo Macrophages
22	t	+	-	+	+++	+++	+	+	ND	-	
	d		+	++	+++	++	+	+	ND	-	
	t ^1^			100							11,318
	d ^1^			99.8				0.2			213,552
23	t	-	-	+	+++	++	-	-	ND	-	
	d		+	++	+++	+	+	-	ND	-	
	t ^1^			100							9107
	d ^1^			99.8					0.2		37,836
24	t	+	-	+	+++	++	+	-	ND	-	
	d		++	+++	++	++	-	-	ND	-	
	t ^1^			99.5		0.1	0.1	0.3			52,812
	d ^1^			99.6		0.1		0.3			195,176
25	t	+	-	+	+++	+++	+	+	ND	-	
	d		++	++	++	+	-	-	ND	-	
	t ^1^			96.4				3.6			28,368
	d ^1^			98.3				1.7			42,048
26	t	+++	-	+	+++	+++	++	+	ND	-	
	d		++	++	++	+	+	-	ND	-	
	t ^1^			100							10,161
	d ^1^			99.9				0.1			57,852
27	t	+	+	++ ^3^	+++	+++	++	-	ND	-	
	d		+++	+++ ^3^	+	+	-	+	ND	-	
	t ^1^			99.7 ^3^		0.1		0.2			22,158
	d ^1^			87.4 ^3^		0.1		0.2	12.3		467,292
28	t	-	+	+ ^3^	+++	+++	+++	-	ND	+	
	d		+++	+++ ^3^	+	+	+	+	ND	-	
	t ^1^			96.9 ^3^				3.1			9856
	d ^1^			94.1 ^3^				5.9			67,437
29	t	-	+	+ ^3^	+++	+++	+	+	ND	-	
	d		+++	+++ ^3^	+	++	-	-	ND	-	
	t ^1^			97.7 ^3^				2.3			8466
	d ^1^			95.7 ^3^				4.2	0.1		24,528

Cell number on histological sections: (+++), numerous; (++), moderate; (+), small; (-), none; ND, not determined. ^1^ Data are presented as the percentage of the number of cells of a particular type out of the total number of cells examined in ex vivo culture. ^2^ After Ziehl-Neelsen staining. ^3^ Smoker’s alveolar macrophages.

## Data Availability

The datasets generated for this study are available on request to the corresponding author.

## Supplementary Materials

The following are available online at https://www.mdpi.com/article/10.3390/ijms22073452/s1, Figure S1: Different lung specimens, such as tuberculoma walls (t) and lung tissues distant from tuberculomas (d), are simultaneously used in the study of the TB patients’ resected lungs, Figure S2: Acid-fast *M. tuberculosis*-infected alveolar macrophages are difficult to detect both in fibrotic tissue of tuberculoma walls (t) and in the alveoli of lung tissues distant from tuberculomas (d), but these cells are easily found in the ex vivo cell cultures obtained from the lung specimens of the same TB patients, Figure S3: Different characteristics of tuberculoma walls on the histological sections are presented for some TB patients, Figure S4: (A–D) Single *Mtb* or *Mtb* in colonies, including those with cording morphology, are detected in the pro-inflammatory and bactericidal markers-positive and markers-negative alveolar macrophages in the ex vivo cell cultures obtained from the lung tissues distant from tuberculomas for the TB patients. (E) The colonies of acid-fast *Mtb* with cording morphology in alveolar macrophages obtained from the tuberculoma wall (t) are similar to colonies of *Mtb* in host cells obtained from the lung tissue distant from the tuberculoma (d) for the same TB patient.
