# Supplementary material for: Mycobacterium tuberculosis Load in Host Cells and the Antibacterial Activity of Alveolar Macrophages Are Linked and Differentially Regulated in Various Lung Lesions of Patients with Pulmonary Tuberculosis
Source: Int J Mol Sci. 2021 Mar 26;22(7):3452. doi: 10.3390/ijms22073452 (PMC8037353; doi:10.3390/ijms22073452)
Supplement: Supplementary file 1 [file ijms-22-03452-s001.pdf]

Supplementary Materials for

Mycobacterium tuberculosis Load in Host Cells and the Antibacterial Activity of Alveolar Macrophages are Linked and Differentially Regulated in Various Lung Lesions of Patients with Pulmonary Tuberculosis

Elena G. Ufimtseva[1,*], Natalya I. Eremeeva[2], Tatiana V. Umpeleva[2], Diana V. Vakhrusheva[2] and Sergey N. Skornyakov[2]

[1] *Laboratory of Medical Biotechnology, Research Institute of Biochemistry, Federal Research Center of Fundamental and Translational Medicine, 2 Timakova street, 630117 Novosibirsk, Russia*

[2] *Scientific Department, Ural Research Institute for Phthisiopulmonology, National Medical Research Center of Tuberculosis and Infectious Diseases of Ministry of Health of the Russian Federation, 50 XXII Partsyezda street, 620039 Yekaterinburg, Russia*

*Corresponding author. Tel. +7 9134806211. E-mail address: ufim1@ngs.ru (E.G. Ufimtseva)

Figure S1

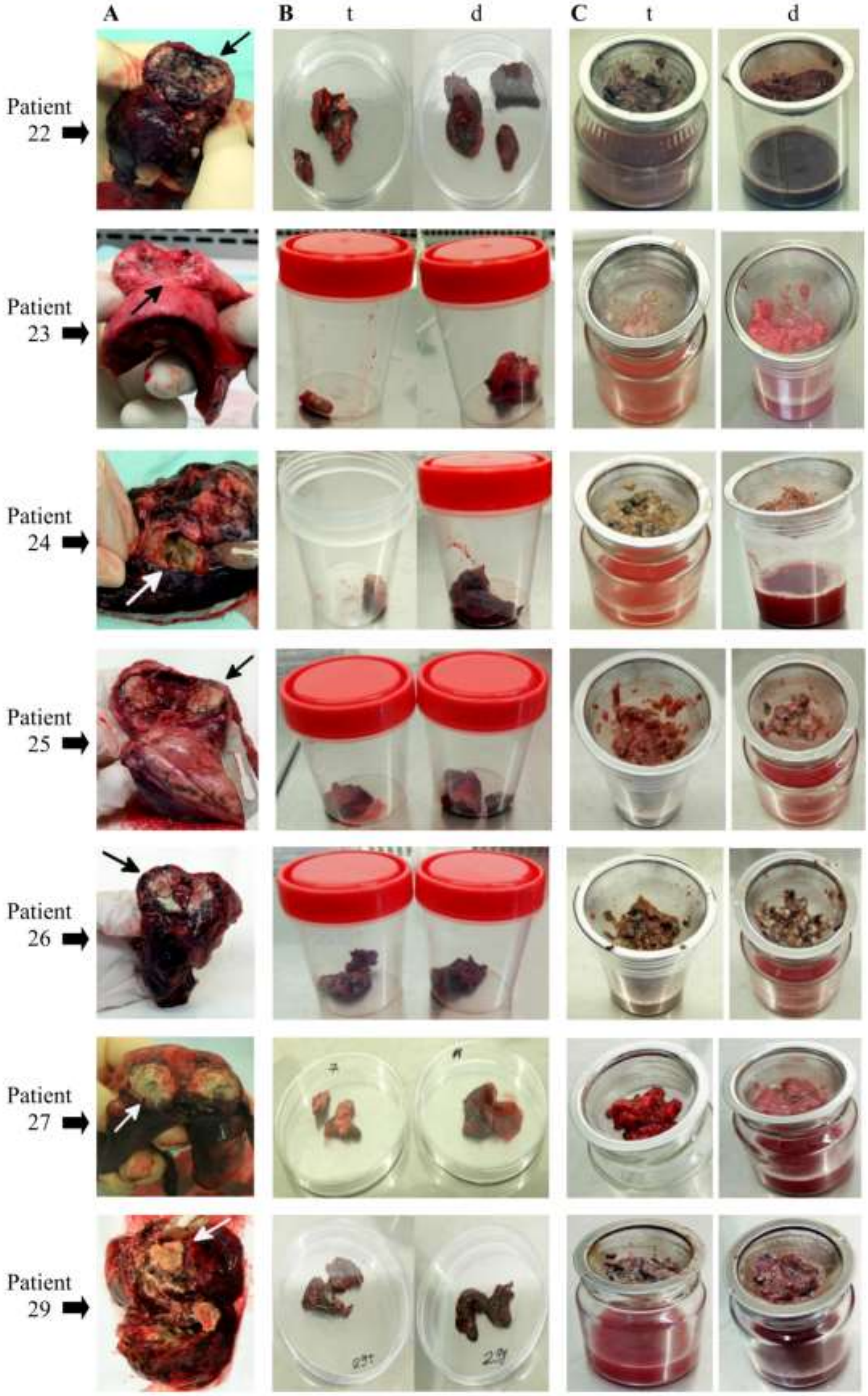

A
B
t
d
C
t
d
Patient 22
Patient 23
Patient 24
Patient 25
Patient 26
Patient 27
Patient 29

Figure S1. Different lung specimens, such as tuberculoma walls (t) and lung tissues distant from tuberculomas (d), are simultaneously used in the study of the TB patients' resected lungs. (A) Lung parts with caseous necrotic tuberculomas surgically removed from TB patients 22–27 and 29. Tuberculomas are indicated by black and white arrows. (B) Tissue specimens with tuberculoma walls (left box) and some parts distant from tuberculomas (right box) obtained from (A) the resected lungs for each TB patient. Petri dishes (for patients 22, 27, and 29) and plastic containers (for other patients) are 10 cm and 4.5 cm in diameter, respectively. (C) In the sieves, small granulomatous lesions in the fibrous capsule obtained from (B) the lung specimens (the wall of tuberculomas and the lung tissues distant from tuberculomas in the left and right panels, respectively) and separated from the cell suspensions containing alveolar macrophages and other leukocytes.

Figure S2

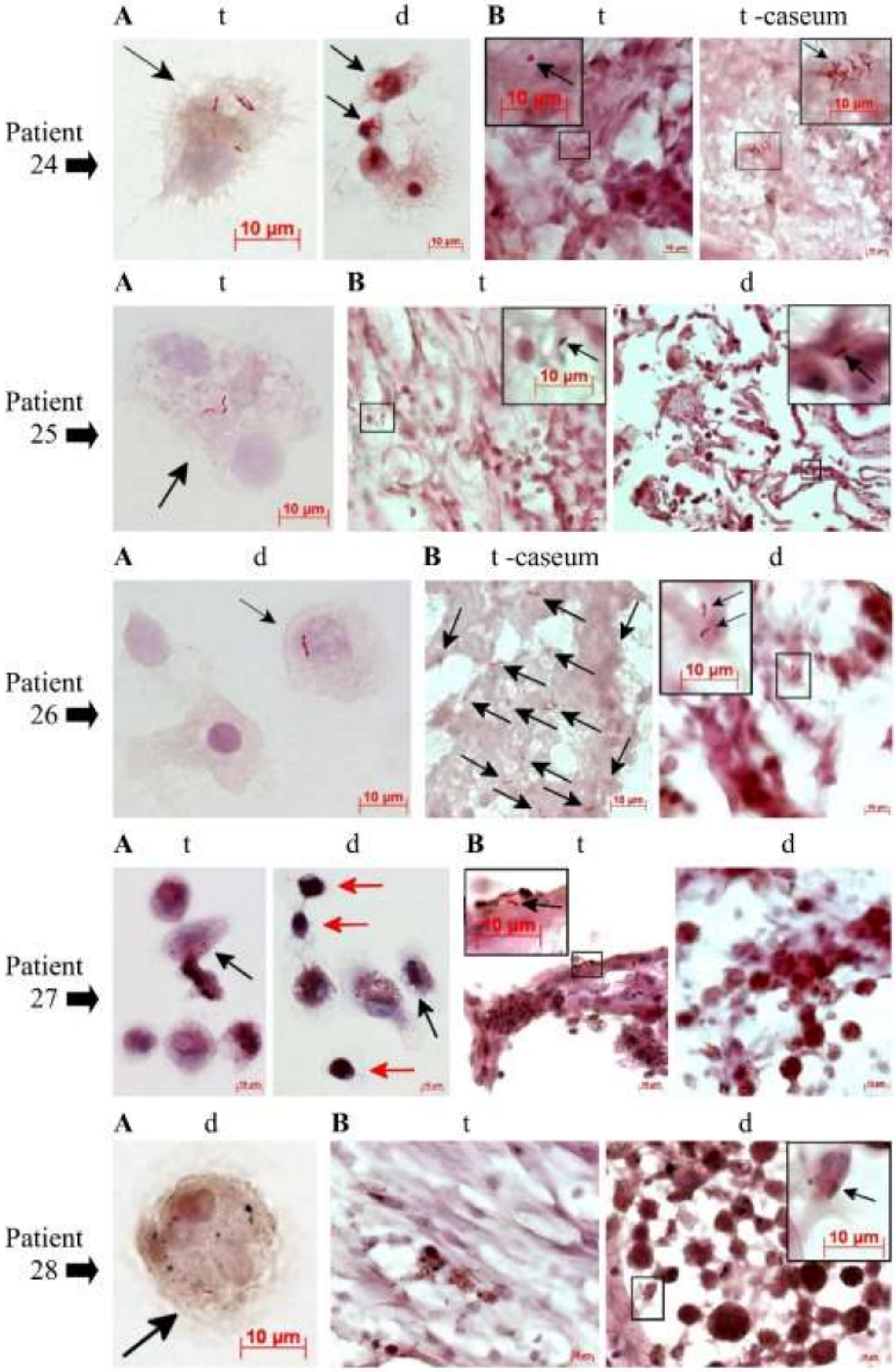

Figure S2. Acid-fast *M. tuberculosis*-infected alveolar macrophages are difficult to detect both in fibrotic tissue of tuberculoma walls (t) and in the alveoli of lung tissues distant from tuberculomas (d), but these cells are easily found in the *ex vivo* cell cultures obtained from

the lung specimens of the same TB patients. (A) Alveolar macrophages obtained from the lung tissues of TB patients 24–28 and stained by the ZN method after *ex vivo* culture for 18 h. (B) Representative images of histological sections obtained from the lung tissues and stained by the ZN method. Close-ups of the parts of these images with alveolar macrophages containing acid-fast *Mtb* are shown in the upper (left or right) corners. (A) Red arrows point to dendritic cells. (A, B) Black arrows point to acid-fast *Mtb* (as single and as colonies, whether cording or not) in alveolar macrophages and in the caseum of tuberculomas. The scale bars are (for patient 25, B, right panel) 20 μm and (A; B, other panels and enlarged views) 10 μm each.

Figure S3

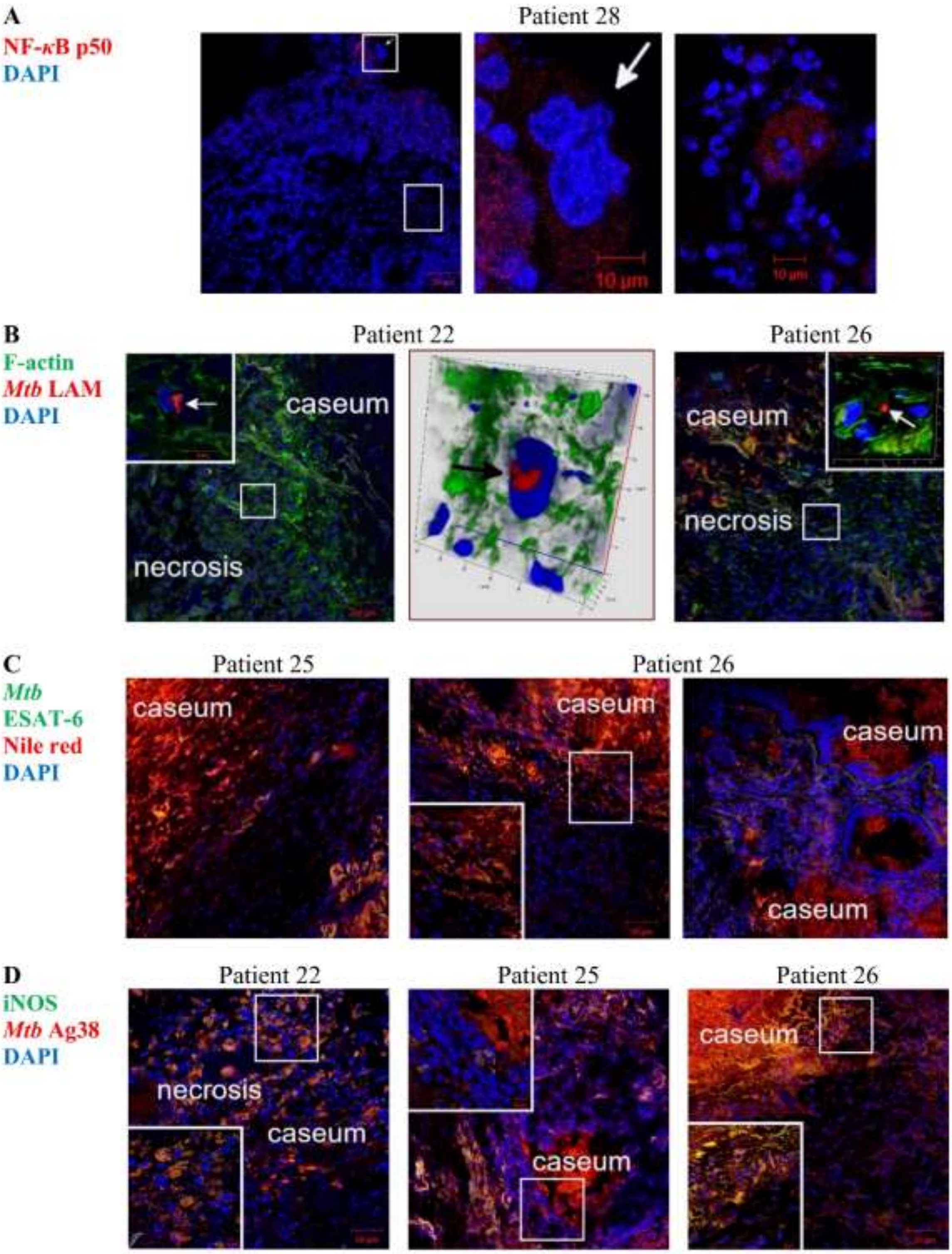

Figure S3. Different characteristics of tuberculoma walls on the histological sections are presented for some TB patients. (A) Close-ups of the parts of the image with an NF-κB p50-stained (red signal) tissue section on the left panel are shown in the central and right panels with a megakaryocyte and neutrophils, respectively. White arrows point to the megakaryocyte. (B-D)

Representative confocal fluorescent images of tissue sections stained by (B) the Alexa 488-conjugated phalloidin dye (green signal) and antibodies to *Mtb* LAM (red signal), (C) antibodies to *Mtb* ESAT-6 (green signal) and the Nile red dye (red signal), (D) antibodies reacting with human iNOS (green signal) and *Mtb* Ag38 (red signal) demonstrate colocalization of some markers (yellow signal). Close-ups of the parts of these images are shown in the corners. (A-D) Nuclei are stained by DAPI (blue signal). The scale bars are 50 μm for the tissue sections and 10 μm for the enlarged views of them. (B) The colonies of *Mtb* are observed in dead cells characterized by the plasma membrane with cortical actin filaments destroyed. (C, D) The caseum at the central zones and the necrotic areas with the dead cells within tissue surrounding the caseum are stained for the markers examined.

Figure S4

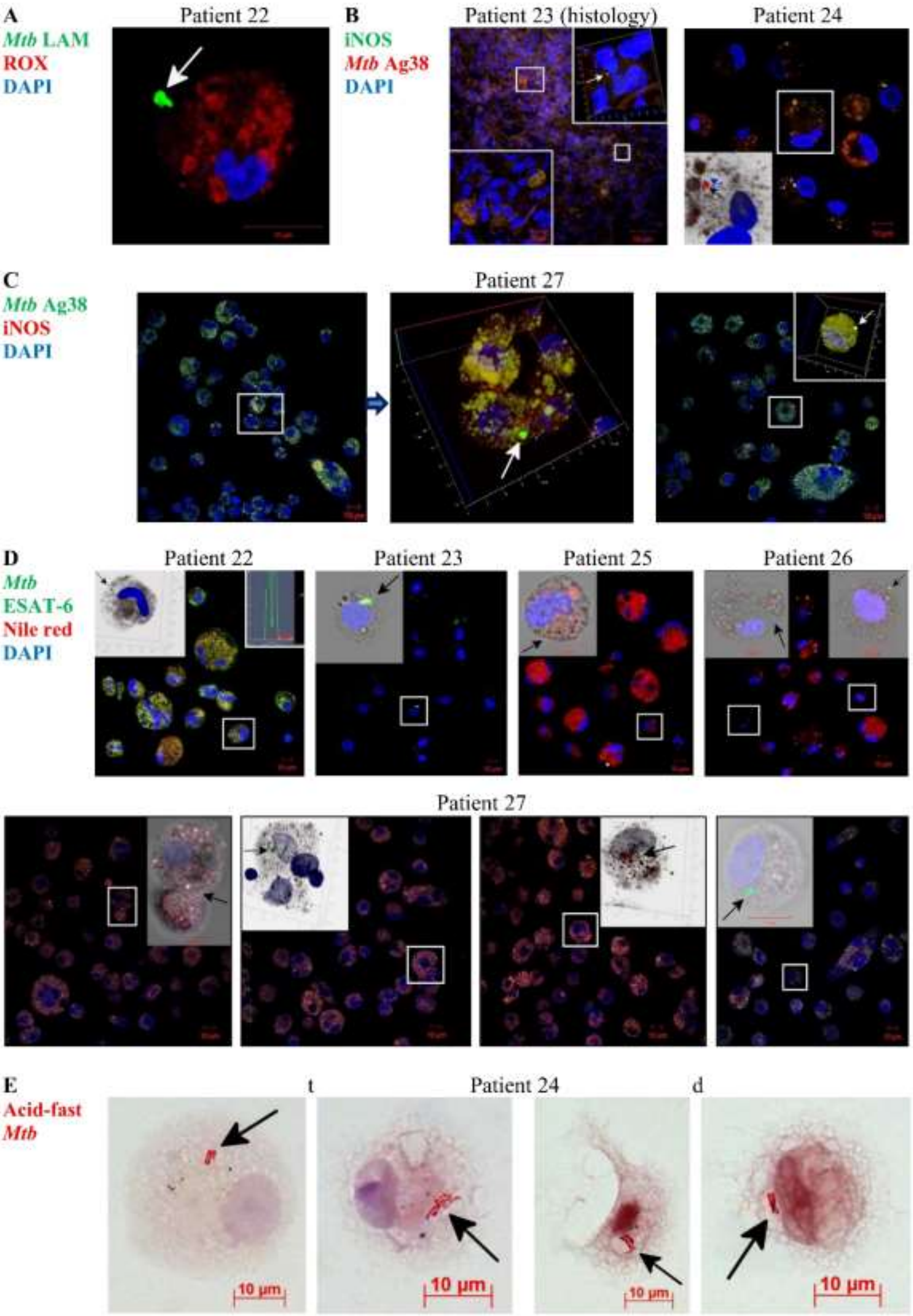

Figure S4. (A-D) Single *Mtb* or *Mtb* in colonies, including those with cording morphology, are detected in the pro-inflammatory and bactericidal markers-positive and markers-

negative alveolar macrophages in the *ex vivo* cell cultures obtained from the lung tissues distant from tuberculomas for the TB patients. (E) The colonies of acid-fast *Mtb* with cording morphology in alveolar macrophages obtained from the tuberculoma wall (t) are similar to colonies of *Mtb* in host cells obtained from the lung tissue distant from the tuberculoma (d) for the same TB patient. (A-D) Representative confocal fluorescent images of alveolar macrophages stained by (A) antibodies to *Mtb* LAM (green signal) and the CellROX Deep Red Reagent (red signal), (B, C) antibodies to human iNOS (green and red signals in (B) and (C), respectively) and *Mtb* Ag38 (red and green signals in (B) and (C), respectively), (D) antibodies to *Mtb* ESAT-6 (green signal) and the Nile red dye (red signal) demonstrate colocalization of some markers (yellow signal). Nuclei are stained by DAPI (blue signal). Close-ups of the parts of these images are shown in the corners. The scale bars are (A-E) 10 μm for the images of the *ex vivo* cell cultures, (B) 50 μm for the tissue section (histology), and 10 μm for the enlarged views of them. (A-E) White and black arrows point to single *Mtb* or *Mtb* in colonies, including those with cording morphology, in alveolar macrophages. (B, right panel) Blue arrow points to single iNOS-stained vesicles in alveolar macrophage. (B-D) 3D or phase-contrasted confocal fluorescent images in the corners. (D, left top panel) A 3D confocal fluorescent image of an alveolar macrophage and a profile image of *Mtb* in colony in the same alveolar macrophage are shown in the upper left and right corners, respectively. (E) Alveolar macrophages stained by the ZN method after *ex vivo* culture for 18 hours.
